# Preoperative rehabilitation and education program for surgery (PREPS): A pilot randomized control trial protocol

**DOI:** 10.1177/17589983251345393

**Published:** 2025-06-20

**Authors:** Rochelle Furtado, Joy C. MacDermid, Dianne Bryant, Kenneth J Faber

**Affiliations:** 1 Department of Rehabilitation Sciences, Faculty of Health Science, 6221 Western University, London, ON, Canada; 2 School of Physical Therapy, Faculty of Health Science, 6221 Western University, London, ON, Canada; 3 Collaborative Program in Musculoskeletal Health Research, Bone and Joint Institute, 6221 Western University, London, ON, Canada; 4 Department of Surgery, 686149 Western University and Roth McFarlane Hand and Upper Limb Centre, St Joseph’s Hospital, London, ON, Canada

**Keywords:** Total shoulder replacement, prehabilitation, exercise, patient education, shoulder arthroplasty surgery

## Abstract

**Background:**

We designed a program for prehabilitation and education before a shoulder replacement (PREPS) that is based on reviews of relevant literature, and is co-designed with preferences of patients recruited from the wait list for a shoulder replacement, and a transdisciplinary healthcare sample. The content and format was integrated in a comprehensive, patient-centered program combining exercise, preparedness for surgery, motivational interviewing, and pain management principles. This patient-centered program has the potential to improve postoperative pain, function, and patient-oriented outcomes, decrease postoperative opioid use and short- and long-term healthcare costs. This pilot randomized controlled trial will evaluate feasibility and satisfaction with our program for individuals undergoing a shoulder replacement.

**Methods:**

Participants (*n* = 90) undergoing a shoulder replacement will be randomized into (1) a 6-week self-directed online pre-rehab and education program, (2) a 6-week online pre-rehab and education program with a therapist to monitor progression of PREPS or (3) a standard of care group. The PREPS program intervention groups will be delivered virtually with an online program of modules and a written handbook. Feasibility outcomes include recruitment rate, adherence, content acceptability, study acceptability, outcome measure completion rates and treatment fidelity. Outcomes will be assessed at baseline and 1 day before surgery, then post-operatively at 6 weeks and 3,6 and 12 months.

**Discussion:**

The proposed project will include the feasibility testing of a prehabilitation and education program with potential to improve surgical outcomes for shoulder replacement patients. Results of this study will provide the foundation for a future fully powered multicenter trial.

**Trial Registration:**

NCT05965986.

## Introduction

A total shoulder arthroplasty (TSA) is a standard operative treatment for a variety of glenohumeral joint conditions, such as shoulder osteoarthritis.^1^ Individuals who experience continued shoulder pain and loss of function, despite conservative management, often undergo a TSA. The overall outcomes of a TSA are quite good with respect to restoring function of the glenohumeral joint,^2^ which is attributed to many factors, including severity of the underlying pathology, surgical technique, and adherence to post-surgery rehabilitation.^1–3^

While the outcomes of TSA are favourable, the surgical procedure can be stressful for patients and their caregivers. Previous research has shown that patient education prior to surgery can lead to better postoperative outcomes and experience for the individual.^4–6^ Education prior to surgery allows sufficient time to process information, or to share information with caregivers and may set more realistic expectations and support less decisional conflict for patients. The concept of preoperative education has been used to increase patient knowledge and behavior change across various surgical procedures.^4–6^

While elective orthopedic surgeries have adopted the concept of preoperative education, there remains a lack of consensus around the key content, delivery, timing of administration and expected outcomes. Further, there is a large amount of literature focused on patient education in lower limb joint replacement surgeries, but a lack of literature addressing upper limb replacement surgeries: the generalizability of treatment effects is unclear. Further, the COVID-19 pandemic uncovered the need for alternative delivery models that maintain social distancing, such as virtual programs. A remote preoperative education program can address barriers to access, ensure information is consistently communicated, and provide individuals an option for repeated reviewing as desired and/or needed.^7–9^

We have developed a comprehensive, patient-centered program that combines exercise, preparedness for surgery, motivational interviewing, and pain management principles.^10,11^ The program is titled Pre-Rehab and Education Program before Surgery (PREPS). The intervention was developed in collaboration with patient partners on the wait list for a shoulder replacement, a transdisciplinary healthcare team (i.e., surgeons, physiotherapists, occupational therapists, and rehabilitation researchers), and current scientific literature. This patient-centered prehabilitation program has the potential to improve postoperative pain, function, and patient-oriented outcomes, and decrease postoperative opioid use and long-term healthcare costs.

Prior to testing the effectiveness of the intervention in a fully powered randomized controlled trial (RCT) we aim to complete a pilot randomized controlled trial to test the feasibility of the intervention and study protocol. Primary feasibility outcomes will be recruitment and retention of patients, given the challenges when sampling an older adult population. Additionally, we anticipate some technological challenges given the intervention will primarily be delivered virtually.

The study will aim to answer the following research questions:

1. What is the acceptability and satisfaction with the PREPS program for patients undergoing a total shoulder replacement?
2. How feasible is the PREPS program in terms of recruitment rate, adherence rate, retention rate, and participant burden?
3. What outcome measures tested in the feasibility trial should be included within the full RCT?

## Methods and analysis

### Study design

This study describes a design of a single center, parallel-group RCT (1:1:1 ratio) to be conducted at the Roth|McFarlane Hand and Upper Limb Centre (HULC) in London, Ontario. HULC is a tertiary care center specializing in the management of complex upper limb disorders and conditions. HULC sees over 40,000 patients per year, and is one of the leading sites for shoulder replacement surgeries in Ontario. The program itself will take place online, with patients being able to engage with the program at their homes.

### Trial design

The pilot study will be a single-blinded randomized trial with three intervention groups. Participants will be randomized to either:

1. A group consisting of the PREPS program 6 weeks before surgery
2. A group consisting of the PREPS program 6 weeks before surgery + therapist following
3. A group consisting of the current standard of care at HULC, which is a WebEx (online synchronous) pre-operative education class lead by a physiotherapist and occupational therapist.

There will be a one-to-one allocation ratio. Groups will be stratified by biological sex (male/female) and replacement type (reverse or anatomical), using a web-based randomization software. The sample size was designed based on multiple papers and criteria for pilot studies and to provide sufficient data to pilot our disaggregated sex/gender-based analyses (SGBA) plan.^12^ Given the predominance of women in this population, we will specifically recruit men to ensure that at least 40% of participants are men, to support SGBA. Allocation will be concealed until eligibility is fully determined. All outcome measures will be performed by a X evaluator.

### Participants

We will recruit volunteers over the age of 18 years, who have been consented for a shoulder replacement surgery at the HULC, to fulfill a sample size of 90 participants, with 30 in each arm. The sample size is based on current recommendations for pilot studies, objectives of this study, and feasibility of being able to complete recruitement of the full powered trial in 2 years. Since our sample size is 168 in the full powered trial, we aim to collect half of this in 1 year. See below for full trial sample calculation.

#### Inclusion criteria

• Over the age of 18 years
• Patients who have consented for a total shoulder arthroplasty or reverse shoulder arthroplasty surgery at HULC
• Date of surgery must be no sooner than 9 weeks, to ensure time for baseline measures
• Speaks and reads in English
• Able to provide informed consent
• Willing to participate in study procedures for at least 1 year post-surgery

#### Exclusion criteria

• Patients who have consented for a hemi-arthroplasty surgery
• Any contraindications to exercise
• Patients with medical conditions that preclude participation in the sessions or follow-up

### Patient and public involvement

**Figure 1.**
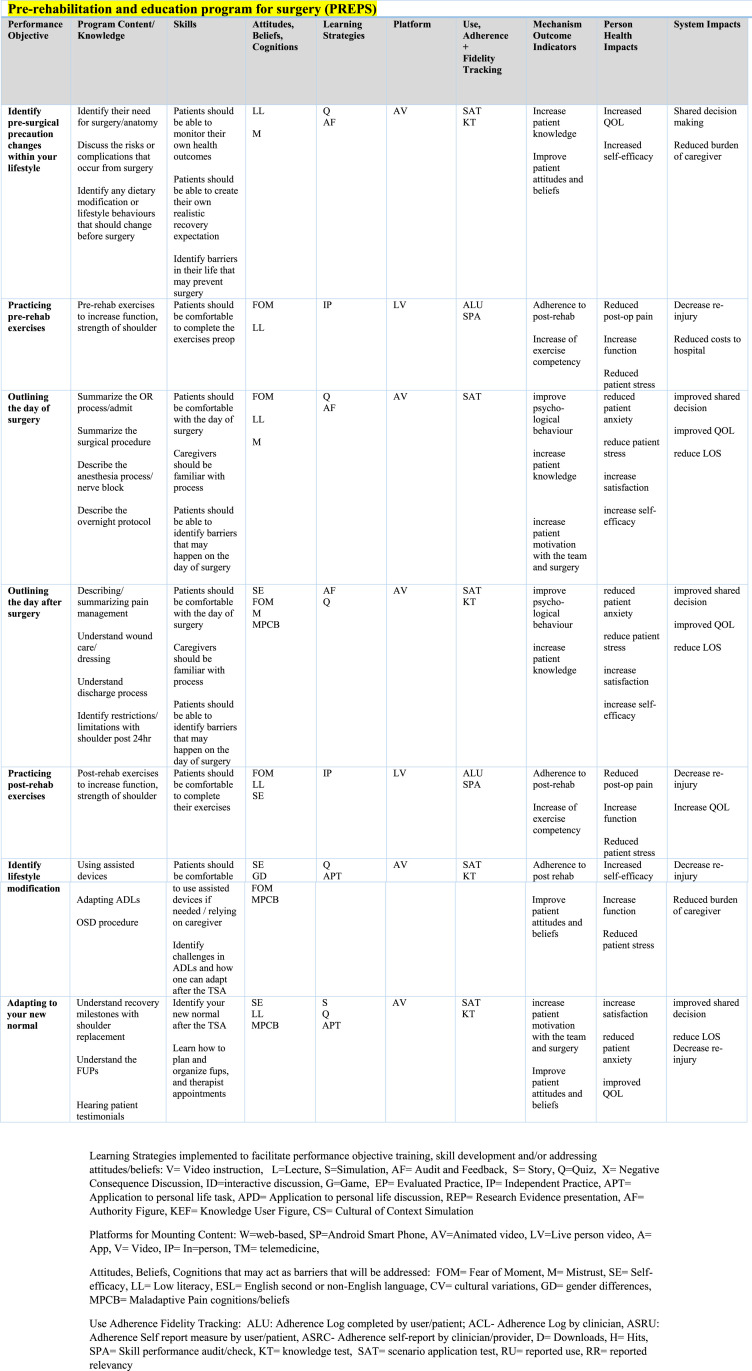
Intervention mapping for PREPS program.

Previous work was done by our group to survey patients on the wait list for a shoulder replacement surgery and clinicians with experience treating patients undergoing a shoulder replacement to better understand preferences of implementing a preoperative online program. Figure 1 details the intervention map used to create PREPS. Patient partners were consulted about the online program to inform feasibility, patient burden and relevance. Future sub-studies will investigate patient engagement and usability with the intervention.^21,22^

### Recruitment procedure

**Figure 2.**
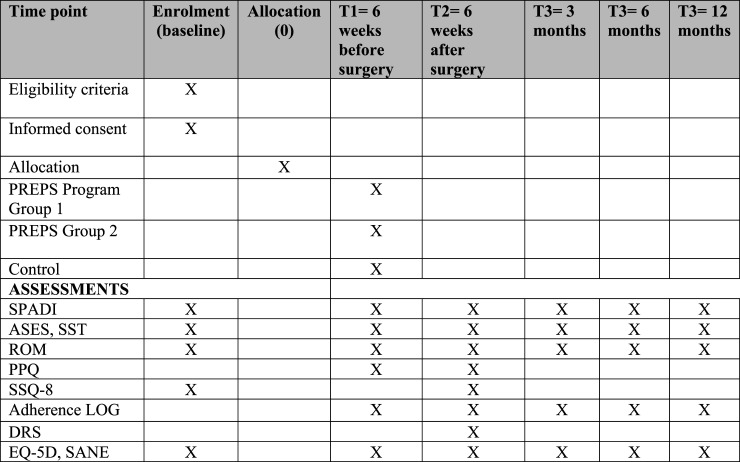
The schedule of enrolment, interventions and assessments for PREPS trial.

The overall flow of the trial is described in Figure 2. Patients who are referred to the HULC and meet the eligibility criteria, will be invited to participate in the study. Clinicians and research staff are trained on the recruitment procedure to maximize the recruitment rate. Information and initial recruitment about the study will be made by the attending surgeon. If patients are interested, a research associate will follow up with the patient to provide additional study details and/or answer questions. Patients will have sufficient time to review study related documents before providing written consent to be enrolled in the study.

### Randomization and concealment of allocation

Patients who are eligible and have consented, will be randomized by a 1:1:1 before their baseline assessment. *A priori*, the principal investigator (JM) will prepare the randomization sequence through computer- generated software, stratifying for sex and implant type, to ensure groups are balanced on these important prognostic factors. Allocation assignments will be sealed in a numbered, opaque envelope prepared by the research associate. Envelopes will be accessed only after patient consent has been obtained. 

### Blinding

The lead student investigator will not be blinded to participant allocation, and so any follow up visits and testing will be conducted by a blinded research assistant. The therapist who will follow patients randomized to Group 2 will not be blinded as they will only have access to patients in Group 2.

### Intervention

PREPS is a 6-week multimodal program that consists of a) a website with video modules, containing both exercises and education on the shoulder replacement journey, as well as b) an education booklet replicating the online information. This multimodal format allows for increased accessibility of information for an older adult population. The program will be completed within a participant’s home, and on their own time over the course of 6 weeks. Patients will be able to replay videos as many times as needed. Interactive quizzes will be displayed throughout the program to capture learning.

#### Exercise content in PREPS

Participants will have the opportunity to complete a short exercise program consisting of five exercises (Hand behind back, forward flexion, ball to the wall, shoulder blade squeezes and pendulums) that are appropriate for pre-surgical rehabilitation. Participants will learn these exercises on screen and then can follow along to complete them. Each exercise video is 2 minutes long. Participants should complete 10 reps, 3 sets of each exercise in the video. This would require approximately 15 minutes from start to finish if a patient completes all five videos. Participants will record their progress on a program sheet, indicating the number of sets and repetitions completed, and any modifications they made during the session.

#### Education content in PREPS

Participants will be guided through an education program that will cover a variety of 7-8 topics regarding before and after surgery preparation. Individuals will have flexibility as to when and how often they review material. The participants will be directed to complete on-screen interactive quizzes to re-enforce learning. Participants are encouraged to pose any questions regarding the material within the education modules to the research team for further clarification. Research assistants will keep a log of calls and questions to inform potential additions or changes to the existing material. Participants will be expected to complete a log to reflect how much of the program they completed and how often each module was reviewed. This will be accessed in their workbooks.

#### Therapist involvement

For participants in Group 2, at the end of weeks 1 and 5, a physical therapist will contact the patient by phone to review the exercise performance. They will implement a teach-back type approach to assess adherence/ health literacy, and address questions or concerns patients have with the education or exercise material. The therapist may suggest adjustments to the exercise program or additional resources to address individual needs/concerns. Phone calls should take approximately 15-30 mins each. To inform treatment fidelity, the therapist will complete a checklist following each call.

#### Time

The intervention groups will be asked to participate in the pre-operative program (6 weeks) before surgery. After surgery, they will not be required to continue with the PREPS program. Standard practice post-operative rehabilitation will be continued with all participants. Patients have the choice on where they want to receive post-surgery rehabilitation but, we will ask participants to track their activity and adherence to physical therapy through a log for 6-month post-operatively.

#### Comparator

The control group will not receive the new preoperative program. They will participate in the standard of care at the HULC, which consists of regular appointments with their surgeon, check-ins with the pre-admit staff at HULC, and the webinar class with a physiotherapist and occupational thereapist. This webinar class, is a one time class with both anatomical and reverse shoulder replacement patients: caregivers are also welcomed. The class consists of two parts, part one focuses on the anatomy of the shoulder, some aspects of the surgurical procedure and anaesthesia information led by a physiotherapist. Part two focuses on how to prepare your environment for after surgery such as clothing, bathing and sleeping, lead by an occupational therapist. The class is an hour long, on an online synchronous platform, and runs monthly. Patients are requested to attend the class once prior to surgery.

#### Data collection

A study manual describing the operating procedures, consent forms, and data forms are in the PREPS Study Manual, accessible at HULC. All data collection assessments will be collected on paper and entered into a de-identified database on a secure and encrypted hospital computer. This information will only be accessible to authorized study personnel.

### Outcomes

All participants, regardless of group will complete all study visits (consisting of 2 pre-operative visits [baseline and one day before surgery] and post-operative visits at 6 weeks, 3 months, 6 months and 1 year). At baseline, we will collected demographic characteristics such as: sex, height, weight, reason for surgery, symptom duration, affected shoulder, and dominant hand. In the pilot study, we will test all outcomes to determine which measures will be moved forward to the full study.

#### Feasbility outcomes

• Recruitment Rate: We will proceed with the larger trial if we are able to recruit 90 participants within 24 months, and if at least 50% of eligible participants consent to participate.
• Adherence: Adherence to the education program will be considered adequate if 80% of all participants in the intervention groups complete the full online program and report it on the log. Exercise programs will be considered adequate if 60% of participants in the intervention groups report exercise at least 3 times per week.
• Content Acceptability: Content acceptability will be assessed using item 7 on the Surgurical Satisfaction Questionnaire, where acceptability will be considered adequate if 60% of participants were satistifed with the treatment (score of atleast 4/5 on item 7).
• Study Acceptability: Study acceptability will be assessed using item 10 on the Surgurical Satisfaction Questionnaire, where acceptability will be considered adequate if 60% of participants were satistifed with the study (score of atleast 4/5 on item 10).
• Treatment Fidelity: If 80% of the content was covered in 80% of phone calls fidelity checklist assessment, fidelity will be considered acceptable.

#### Outcomes to be included within the full study

The following outcomes listed below will be included in the pilot study since participants in the pilot may be rolled into the full study (see Table 1). The primary outcome in the full study will be the SPADI and secondary outcomes will include:

• *General health status* as measured by EQ-5D global health^13^ and Single Assessment Numeric Evaluation^14^
• *Shoulder specific function tests*: American Shoulder and Elbow Surgeon’s Score,^15^ Shoulder Pain and Disability Index,^16^ Simple Shoulder Test^17^
• *Patient satisfaction*: Surgical Satisfaction Questionnaire (SSQ-8)
• *Patient Anxiety *as measured by the Decisional Regret Scale,^18^ and Patient Anxiety Symptom Scale (PASS)
• *Adherence to rehabilitation* as recorded in the patient log (see Table 1)
• *Performance measures*: goniometric measures of shoulder range of motion

**Table 1.** DRS, decisional regret scale; SPADI, shoulder pain and disability index; SANE, Single assessment of the numeric evaluation; PPQ, patient participation questionnaire; SSQ-8, surgical satisfaction questionnaire; SAQ, surgical anxiety questionnaire.

Variable/outcome	Hypothesis	Outcome measure	Method of analysis
Recruitment	15-16/ month And 50% eligible to participate	Research log	Percentages reported

Adherence	80% of all participants in the intervention groups complete the PREPS program, and 60% report exercise at least 3 times per week	Patient log	Percentages reported
Content acceptability	60% of participants found the treatment useful and helpful on item 7	Surguical satisfaction quesionnaire	Means and standard deviation reported
Study acceptability	60% of participants found the treatment delivery acceptable, and reported being likely to use this treatment again and recommend it to others, on item 10	Surguical satisfaction quesionnaire	Means and standard deviation reported
Treatment fidelity	If 80% of phone calls and recorded notes were documented on the checklist by the therapist	Therapist checklist	Means and standard deviation reported

The majority of assessments will occur at baseline, day before surgery, and post-surgery at 6 weeks, 3 months, 6 months and 12 months. With the exception of some measures, as detailed in Figure 1, of the schedule throughout the research study.

#### Data management and monitoring

Data collection and management procedures have been approved by the Western University Research Ethics board (#120163) and Lawson Health Research Board (R-23-550). Identifiable data will be kept separate from the main data files and will be accessible to authorized study team members only. Data entry and coding of the de-identified data will be conducted by a blinded research assistant. If any adverse events occur that are study related, they will be discussed by the research team collectively. 

## Sample size estimation

Our sample size calculation will be based on a formula for analysis of covariance (ANCOVA), with an estimated association between preoperative and postoperative SPADI scores at 3 months.^19^ Correlation of SPADI scores from baseline and 3 months will be determined from pilot trial findings, however, we have estimated a calculation based on current literature of the SPADI in arthroplasty patients.^19,20^

Calculation: N per group = 2((Z_α_ + Z_β_)^2^σ^2^(1-r^2^))/(δ-M)^2^, where:

α = the probability of making a Type I error = 0.05, one-sided test

1-β = the power to detect a difference if one truly exists = 0.80, thus, β = 0.20

δ = MCID (within-group) = 20.6, adjust for between-groups comparisons 20.6*0.5 = 10.3

r = at 3 months = 0.81

σ = 21.3 (SD at 1 year post-operative)^20^

M = 0.2 SD (4.26), margin for non-inferiority (Group 1 v Group (2) and superiority (Group 1 v SOC)

N per group = 2((1.64 + 0.84)^2^ (21.3)^2^(1-0.81^2^))/(10.3-4.26)^2^ = 53 patients per group

The estimate per group will then be increased by 5% to adjust for potential losses over time (*n* = 56/group).

## Plan for statistical analyses

Descriptive quantitative variables will be presented as means and standard deviations and the categorical variables will be presented as percentages. Following pilot study guidelines, criteria to progress from feasibility to definitive trial are listed in Table 2 below. Each primary feasibility outcome measure will be assessed separately, and overall progression will be determined by the worst-performing criterion. If all signals fall into the green zone, we will proceed to the trial without changes. If none of the signals fall into the red zone but at least one falls into the amber zone, the design of the trial procedures will be amended before proceeding to the definitive trial. If at least one signal falls into the red zone, we will not proceed to the definitive trial without making significant improvements to protocol and re-assessing feasibility.

**Table 2.** Progression criteria for feasibility.

	Proceed	Proceed with protocol amendment	Significant protocol amendment
Recruitment *N* = 90 in 12 months	15-16/ month and 50% eligible to participate	13-14/ month and 40% eligible to participate	<12 /month and 30% eligible to participate
Adherence	• 80% of all participants in the intervention groups complete the online program, and 60% report exercise at least 3 times per week	• 60% of all participants in the intervention groups complete the online program, and 40% report exercise at least 3 times per week	• 25% of all participants in the intervention groups complete the online program, and 25% report exercise at least 3 times per week
Content acceptability	• 60% of participants found the treatment useful and helpful with a score of 7/10	• 40% of participants found the treatment useful and helpful with a score of 7/10	• <40% of participants found the treatment useful and helpful with a score of 7/10
Study acceptability	• 60% of participants found the treatment delivery acceptable, and reported being likely to use this treatment again and recommend it to others with a VAS score of 7/10	• 40% of participants found the treatment delivery acceptable, and reported being likely to use this treatment again and recommend it to others with a VAS score of 7/10	• <40% of participants found the treatment delivery acceptable, and reported being likely to use this treatment again and recommend it to others with a VAS score of 7/10
Fidelity of treatment	• If 80% of phone calls and recorded notes were documented on the checklist by the therapist	• If 60% of phone calls and recorded notes were documented on the checklist by the therapist	If <40% of phone calls and recorded notes were documented on the checklist by the therapist

## Ethics and dissemination

This trial is designed to be more pragmatic than exploratory, in the hopes of the intervention to be adopted into practice and eventually expanded into other hospitals within the country. This study will be the first to address a program for both reverse and total shoulder replacement patients, as usually studies focus on lower limb replacements, and if they incorporate shoulder replacements, it is only on total anatomical shoulder replacements. Reverse and anatomical shoulder replacements vary in post-surgical outcomes; indicating the need to make sure the program is able to educate all patients, regardless of implant type.

This study was designed to be presented in English which may limit the external validity of this study. During our previous work, results indicated that majority of people receiving a total shoulder replacements at the HULC were English speaking. Future iterations of this work however can include translations to target a larger population.
